# Nucleic Acid Delivery with Red-Blood-Cell-Based Carriers

**DOI:** 10.3390/ijms22105264

**Published:** 2021-05-17

**Authors:** Giulia Della Pelle, Nina Kostevšek

**Affiliations:** 1 Department for Nanostructured Materials, Jožef Stefan Institute, Jamova Cesta 39, 1000 Ljubljana, Slovenia; nina.kostevsek@ijs.si; 2 Jožef Stefan International Postgraduate School, Jamova Cesta 39, 1000 Ljubljana, Slovenia

**Keywords:** drug-delivery system, biomimetic materials, gene therapy, erythrocytes

## Abstract

Gene therapy has the potential to become a staple of 21st-century medicine. However, to overcome the limitations of existing gene-delivery therapies, that is, poor stability and inefficient and delivery and accumulation of nucleic acids (NAs), safe drug-delivery systems (DDSs) allowing the prolonged circulation and expression of the administered genes in vivo are needed. In this review article, the development of DDSs over the past 70 years is briefly described. Since synthetic DDSs can be recognized and eliminated as foreign substances by the immune system, new approaches must be found. Using the body’s own cells as DDSs is a unique and exciting strategy and can be used in a completely new way to overcome the critical limitations of existing drug-delivery approaches. Among the different circulatory cells, red blood cells (RBCs) are the most abundant and thus can be isolated in sufficiently large quantities to decrease the complexity and cost of the treatment compared to other cell-based carriers. Therefore, in the second part, this article describes 70 years of research on the development of RBCs as DDSs, covering the most important RBC properties and loading methods. In the third part, it focuses on RBCs as the NA delivery system with advantages and drawbacks discussed to decide whether they are suitable for NA delivery in vivo.

## 1. Introduction

Despite some successes, the effective treatment of most cancers remains problematic for several reasons: the lack of drug specificity and the drug-resistant properties of cancer cells and metastases that are a major contributor to the deaths of cancer patients. In recent years, the development of sophisticated genomic, proteomic and bioinformatics techniques have made it possible for us to glimpse the intricate interplay of numerous cellular genes and regulatory genetic elements responsible for cancerous phenotypes. As a result, one of the key advances in this new era is gene therapy, which focuses on the therapeutic delivery of nucleic acids (NAs), such as the small interfering RNA (siRNA), antisense oligonucleotides (ASO) or plasmid DNA (pDNA), into a patient’s cells to replace the abnormal or absent genes with healthy ones. However, NAs present challenges related to stability and in vivo delivery that need to be overcome [1,2]. For example, NAs are prone to degradation by enzymes, are rapidly excreted from the body and negatively charged, which limits their uptake in target tissues and cells [3]. The introduction of large quantities of double-stranded RNA in mammalian cells also activates interferon-related pathways [4]. Therefore, safe delivery systems, allowing the prolonged circulation and expression of the administered genes in vivo must be developed [5]. 

A drug-delivery system (DDS) must carry a sufficient amount of drug to the target site with the most efficient kinetics, avoiding side effects. The DDS should improve the drug’s water solubility, thermostability and reduce susceptibility to degrading enzymes. The ideal DDS must add at least another function to the whole formulation [6], for example, “theranostic” approaches, combining therapeutic and diagnostic abilities [7,8,9]. Currently, most of NAs carriers has a cationic charge in order to maximize stability of the formulation and to enhance cell delivery [10], while there are some concerns about the toxicity; therefore, neutral or mildly anionic materials to entrap or carry NA have begun to be investigated, to ensure a better bio-compatibility and broad the application range [11]. 

Though highly developed and well-characterized, most synthetic DDSs are recognized and eliminated as a foreign substance by the immune system. To overcome this, biomimetic cell-membrane-based nano-carriers were proposed. Disguised by the cell membranes, the drugs, nanoparticles or other active components can act as autogenous material due to their bio-compatibility. Among the different circulatory cells (monocyte, erythrocytes, macrophages, lymphocytes, neutrophils, platelets, leukocyte, dendritic cells, stem cells, and extracellular vesicles), red blood cells (RBCs) are the most abundant and thus can be isolated in sufficiently large quantities to decrease the complexity and cost of the treatment compared to other cell-based carriers [12,13]. Later in this paper, the state of the art for the use of the red blood cells (RBCs) as drug carriers will be presented. Since the delivery of NAs has great promise for the treatment of several diseases, in Section 4 we will focus on the delivery of NAs with both whole RBCs and derived vesicles as their carriers. This is the first systematic review of erythrocytes used as NA-based drug carriers. In addition, the so-far unexploited advantages and the drawbacks will be examined. 

## 2. Evolution of Drug-Delivery Systems (DDSs)

The first DDS was introduced in 1953, based on Spansule^®^ technology [14], while the first incorporation of a drug into non-toxic polymers occurred in 1976 [15], leading to the concepts of “sustained release” and “controlled release”. The number of papers and achievements has grown exponentially since then [6,16]. For a long time, chemists and pharmacologists searched for, with polymeric, encapsulated or via mechanical devices, a zero order, that is, a totally controlled [17,18,19] pharmacokinetic, to maintain the concentration of the drug. This led to a huge increase in the research of biocompatible polymers and hydrogels. Later it was realized that a zero-order kinetic of release is not necessary to achieve an efficient pharmacology effect, so the idea was abandoned [20]. This led to the development of pH, temperature, light, redox reactions, ultrasound, triggered DDS, made up of various biodegradable materials that promote a secondary effect, for example, immunogenic or anti-inflammatory [21].

The greatest successes have been achieved with nanotechnologies [22] and the re-purposing of old molecules resulting from the COVID-19 pandemic [23,24,25]. Liposome encapsulation is the technique of choice for last-generation DDSs [26]. Liposomes are easy to synthesize and modify to add new features such as a liquid-crystal transition-point shift, membrane rigidity or conjugation with stealth polymers. They represent the most cost-effective nanostructure. Furthermore, liposomal formulations are capable of the so-called EPR effect (enhanced permeability and retention) [27]. Such nanostructures extravasate easily and tend to accumulate in the proximity of tumors due to the leaky nature of tumor blood vessels, rich in fenestrations and poor in smooth muscles. So far, in humans, the effect has only been widely documented for Doxil^®^ [28], while there remains widespread skepticism about the existence of this phenomenon in humans [29]. 

There are only a few drugs approved by the FDA (Food and Drug Administration) that exploit a liposomal DDS [30]: doxorubicin, sold as Doxil^®^ or Caelyx^®^ [28], amphotericin B, sold as AmBisome^®^, and MM-398^®^/Onivyde, a irinotecan-loaded nanoliposome formulation containing 5-fluoro-uracil [31]. Recently, there is the Pfizer-BioNTech Sars-cov-2 vaccine (an mRNA contained in DSPC liposomes, NCT04368728). The Thermodox [32,33] formulation, containing a lysolipid, is actually in phase-III clinical trials for radio-frequency ablation treatment. DSPE-PEG_2000_ is present in a 10:90 lipid ratio, enough to show a “stealth effect”, while MSPC (1–4%) lysolipid ensures a payload release upon heating. Examples of targeted, differentially loaded, coated, liposomes are numerous in the literature [26,34,35]. 

Artificial nanoparticles (polymeric, dendrimeric, liposomal or combinatory approaches) are often recognized as non-self by the immune system [36,37], so compromising both the “non nocere” principle to healthy tissues and the appropriate circulation time, jeopardizing the overall therapeutic effect. The most famous example is the case of polyethylene glycol (PEG), a commonly used excipient, known to shield its cargo from the Reticuloendothelial System (RES) (or Mononuclear Phagocytic System [38,39] It acts as a hapten when bound to protein drugs such as IFNα [40], but there are only a few studies indicating the presence of immunoglobulin M (IgM) against PEG elicited by liposomal PEG formulations [41,42,43,44]. Similarly, an immune reaction is elicited towards polyamidoamine (PAMAM), only when bound to a protein carrier, whatever the identity [45]. Initially, artificial nanoparticles were synthesized to be “ignored” by the macrophages, omitting a thorough characterization of how properties like shape, zeta-potential, and solubility influence the immunogenicity of the carrier [37,46]. After realizing that a totally inert nanocarrier cannot be synthesized, effort was put into the codification of a so-called “nano-bio barrier” [47,48] and bring to a new level the “stealth effect” on RES: bio-mimesis, to enhance drug release and elicit/modulate/avoid immune reactions. For instance, newly developed poloxamers like Pluronic^®^ block copolymers [49,50] and the ever-growing application of poly(lactic-co-glycolic acid) (PGLA) [51,52,53], paved the way for a new generation of (tentative) DDS, that is, cell-based systems. So, although liposome formulations remain the most used, cell-based DDSs could soon compete. Here we describe how erythrocytes have been used to encapsulate NAs for use as drugs. We will emphasize the shape, geometry, surface potential, membrane permeability of erythrocytes and nanoparticles, and we attempt to decide whether they are suitable for NA delivery in vivo.

## 3. Erythrocyte-Based DDSs

### 3.1. The Single Erythrocyte: A Complex System

Erythrocytes are the most abundant cells in our bodies, representing 70% of all the cells in the average adult human. They are conserved in the cell lines of all mammals, devoted to oxygen transportation by hemoglobin (Hb), a four-heme-ringed protein they are packed with [54], and of carbon dioxide in the form of bicarbonate ions. They are an approximately 8-µm biconcave disk, anucleated, with a lifespan of 120 days [55]. Their membrane can be considered, from the DDS point of view, a perfect stealth, biocompatible, highly deformable (up to 3 µm in capillaries) [56,57], coating for drugs and diagnostic agents. It is made of phospholipids plus a considerable amount of cholesterol (up to 30% in mass [58]) and shows unique elastic properties due to its cytoskeletal reticulum, composed of different proteins, collectively known as spectrin [59,60], assisted by actin and ankyrin. A different phospholipid content, in the two leaflets of the membrane, is kept constant by flippase and floppase [61,62,63] (the cholesterol is equally distributed) and, furthermore, by calpain (an enzyme hydrolyzing protein membrane anchorages to actin) and gelsolin (that severs actin filaments). All of these enzymes are activated by the presence of calcium ions. Indeed, flippase inactivation leads to membrane budding, leading to natural extracellular vesicles [64,65,66]. Erythrocytes are also the cells in the human body that release the most extracellular vesicles. They lose, by aging, around 30% of their dry mass by budding. However, this is not a completely random process. It is known that the responsibility for this budding lies with the phosphatidylserine’s presence in the outer leaflet and that, probably, clearance towards the membrane attack complex of a complement protein system [67]. The required ATP for membrane-lipid-related reactions is provided by glycolysis Embden- Meyerhof enzymes and it accounts for nearly all the energy expended by an erythrocyte [68,69]. With respect to the elastic properties of the erythrocyte membrane, it was observed that the membrane is able to form tethers in RBCs too [70,71]. As recently reviewed by Ciana [72], erythrocyte exhibits a web-like network of lipid rafts, mostly composed of glycosphingolipids, sphingomyelins, and cholesterol, an anchorage site for spectrin cytoskeleton. So, any contamination with circulating granulocytes, containing proteases and hydrolases, in ex vivo conditions, damages the erythrocyte structure. Erythrocyte’s physiological shape is biconcave, while stomatocytes and echinocytes are pathological three-dimensional structures [73]. The molecular bases of such an arrangement are still unknown, but they probably rely on the different membrane densities between the rim and the dimple of the cell [74] (more details in Section 4.2.1).

Erythrocyte proteome and interactome have been the subject of much research in the past 10 years, leading to discover of a considerable complexity [75,76,77]. Interestingly, it was only during the 1970s that researchers began to realize that RBCs are not only Hb containers. They are also involved in an innate immune response, transferring opsonized particles to macrophages with CR1 protein [78], cytokine storm clearance [79], ROS protection [80], and, of course, initiators of blood clotting by phosphatidylserine (usually confined on the inner leaflet) exposure via a pro-thrombin pathway [81,82]. Erythrocytes also exhibit the so-called, for macrophages, “don’t eat me!” marker CD47 [75,83,84], allowing them to be recognized as self by the RES (specifically by SIRPα receptor on macrophages [78]). They also possess an integral protein, called the decay-accelerating factor, that inhibits complement activation [85]. Their aging process is related to the loss of a segment of the band-3 protein, which is lacking only in senescent cells. This has been known for a long time as the “senescent antigen”. So far, it is the only known auto-immunogenic feature of these cells [86] in the absence of illnesses. A glycocalyx component, sialic acid, also has a huge role in complement complex activation; it usually inhibits complement cascade initiation, but once this begins, it acts as a promoter [87]. Its crystal structure has been recently (2015) elucidated [88].

These properties are an elegant compromise among easy functionalization, loading efficiency, and biocompatibility, with nearly every patient taking care of blood type. The synthesis of erythrocyte ghosts (i.e., erythrocytes depleted of Hb) is easy and quick [5,89]. Anucleated in humans, a non-coding RNA has only recently [90] been discovered in human RBCs. Furthermore, RBCs proteome has been already characterized also with the recent evidence of a nitric oxide synthase in inner leaflet of the cytoplasmatic membrane [91], while a ribonuclease T2 activity was reported by Kabanova et al. in 2009 [92]. Conversely, human erythrocytes are naturally rich in an aspecific RNAse inhibitor, of 31 kDa [93], that, in vitro, removed any RNAse activity in the erythrocyte lysates. Virtually, the RBCs, lacking a nucleus, despite the afore-mentioned conflicting data, could have the advantage of avoiding horizontal gene transfer and, therefore, spreading malevolent mutations. This could be a problem with other cell-type-based carriers. 

Being so ductile, self-marked, capable of natural tropism towards soft tissues, and intrinsically nuclease-free, erythrocytes have been used as a cargo for NAs: the red cell loading modalities as be applied to NA delivery will be described in the next section. 

### 3.2. Development of RBCs as DDSs: Main Techniques

A ground-breaking paper in 1973 [94] a new era for RBC use. Garrett Ihler and his team demonstrated that enzymes up to 180 kDa can be trapped inside an erythrocyte ghost. This term was first used by Hoffmann [95], who also developed a rapid protocol to allow the RBC to release its Hb based on osmotic shocks. This approach became the golden rule for subsequent experimentation with RBC membranes, and led to numerous refinements (already reviewed in the 1980s [96]), as far as they can be carried out in non-pyrogenic conditions. The topic of drug delivery via RBC ghosts was enriched by the Muzykantov group [97]. Multiple techniques have been developed to improve the use of the RBC membrane as a carrier or disguising agent for drugs; they can be divided into three major groups:

- Surface functionalization (also called external loading);
- Coatings for nanoparticles;
- Erythrocyte ghosts and nano-erythrosomes (internal loading);

We will focus on point three, with special attention on NA loading (Table 1). We suggest consulting [23,89,98,99] about points 1 and 2.

### 3.3. Internal Loading Methods 

#### 3.3.1. Hypotonic (pre) Swelling

**Figure 1:**
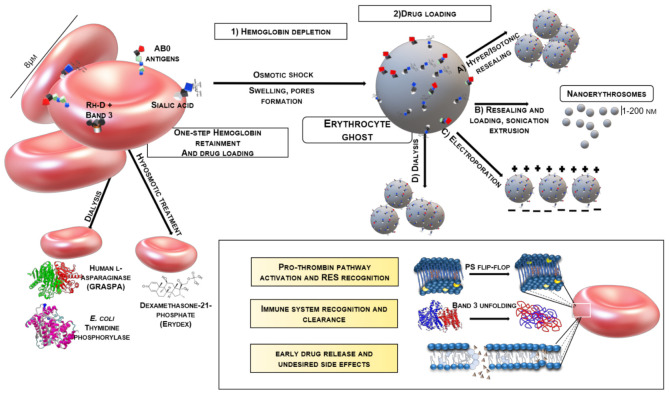
Summary of internal loading methods. Hb can be maintained or not according to the processing procedure and the identity of the cargo. In the inset the most important unwanted effects are shown. The explanation can be found in the text. PDB entries: Human L-Asparaginase 4O0H [113]; *E. coli* thymidine phosphorylase 4LHM [114]; Mouse Band 3 4YZF [88]. Models were created using VMD software.

This cluster of methods relies on the capability of the erythrocyte (full or ghost) to form transient pores of 200–500 nm [107] if the surrounding environment is hypotonic [108,109] (Figure 1). 

The membrane double layer is also subjected to engulfment. When the membrane comes into contact with an isotonic medium, which may or not contain the drug of interest, it reseals, and the erythrocyte returns to its initial volume, while the drug passively flows inside the ghost, where it is trapped. The same approach is widely used to deplete the erythrocyte from Hb [95,110]. Of course, multiple variations of this have been developed, like Hb precipitation with PEG [111], pre-incubation with a hypotonic drug solution and then hypertonic resealing [112], and gradual drug loading [109]. In the only recent paper on the efficient loading of RBC ghosts with plasmidic DNA [100], Larson et al. pre-swelled them in hypotonic phosphate buffer, incubated with plasmidic DNA, than concluded with resealing, adding a hypertonic solution of sodium chloride. The hypotonic pre-swelling approach was commonly used in the 1980s to load RBC ghosts with both RNA and DNA. A complex method for PEG6000-mediated fusion was developed by Wiberg [101], who did not see the RBC ghosts as drug carriers, but as transfecting agents. Earlier, [103] applied a similar techniqueto convey tRNA to a cultured cell. Recently, for active liver targeting, antibody-conjugated, erythrocyte ghosts were loaded via hypotonic swelling with antisense nucleotides [102]. 

The patented red-cell loader (EryDel) is based on hypotonic pre-swelling. It is approved for blood detoxification, loading the patient’s own erythrocytes with a drug such as dexamethasone 21-phosphate and asparaginase [115,116]. These formulations are in clinical trials II/III [117,118].

Hypotonic swelling’s advantages are its blandness, mild conditions (usually room temperature), variability that can be technically applied (also in terms of scalability), and, importantly, the relatively limited damage inflicted to the membrane structure and the mimetic ability of the erythrocyte. Nevertheless, the technique is not suitable for every compound. The molecule should be highly water soluble or must be loaded as a pro-drug (i.e., functionalized with phosphate, citrate, lysin, etc.) to enhance the water solubility, to diffuse inside the swollen erythrocyte and should be chemically inert towards ghost glycocalyx, to avoid external binding and reduce the encapsulation efficiency. 

#### 3.3.2. Dialysis Methods

The principle of dialysis has been applied to erythrocyte ghosts for loading. Most commonly, a bag containing an isotonic solution of erythrocyte ghosts is inserted into 4–10 volumes of a hypotonic solution containing the drug, that passively diffuses inside the bag. The latter cut off being below the molecular weight of the drug [119]. The method was established and refined by Ihler for glucocerebrosidase [120] and recently used by Levene et al. in the successful (currently trial II) encapsulation of [121] thymidine phosphorylase. The GRASPA^®^ formulation, containing for lymphoblastic leukemia is based on dialysis for human L-asparaginase (whose model can be found in fig.1 as elucidated by [113]) loading in erythrocytes [122]. However, despite having passed trial III in 2010, it has not received EMA approval. EryTech developed EryCaps^®^, the French counterpart to the red-cell loader, based on hypotonic dialysis [123] with single-use cartridges. A formulation containing *E. coli* thymidine phosphorilase is also undergoing clinical trials [114] (fig.1). Despite being one of the easiest and most ductile loading methods, there are just three examples of this technique used to load NAs [104,105,106]. This could be due to the high levels of sterility required and nuclease inhibitors needed to be added to the buffer.

#### 3.3.3. Electroporation

Electroporation is the preferred technique to deliver small molecules, proteins or NAs into a cell or a liposome because of the controllability of the physical parameters, the high transfection yields and the preparative procedures [124]. Therefore, the thermodynamic parameters and the density of the created membrane pores depend on the power and duration of the electrical pulse. The phenomenon is also dependent on the membrane’s surface potential, shape, intrinsic permeability and membrane dielectric constant. The electroporation experiments must be specifically optimized for each cell line [125,126]. A number of automatic devices exist [124]. Electroporation has been widely used to lysate [127] and to load erythrocyte ghosts. Usually, the RBCs are suspended from a known hematocrit in an isotonic medium, also containing the drug to be entrapped, subjected to a single pulse of tens of kV/cm (~300 V applied by the electrode) (Figure 1), or more pulses at lower kV/cm, and immediately transferred to a pre-heated isotonic medium to allow resealing [128]. Later, Zimmermann pioneered this approach to load RBCs with methotrexate [129]. The technique was refined for interleukin-2 [130], daunarubicin and doxorubicin [131]. Furthermore, electroporation is probably the most suitable technique for loading NAs inside a DDS. In 2014 the Nair group [13] used electroporation to load mRNA, for a tumor vaccine, with whole blood cells, being composed primarily of erythrocytes and antigen-presenting cells, taking advantage of the presence of RNAse inhibitors in the RBCs. Then, erythrocyte extracellular vesicles (membrane derived and showing similar chemical and physical properties) were used to deliver antisense oligonucleotides [66] to leukemia cells. The EVs were loaded using electroporation with a 25% encapsulation efficiency at 250 V. In addition, plasmid DNA was loaded into erythrocyte ghosts using electroporation [5]. In the same study, they demonstrated that the encapsulation shields the NAs from DNAse I and II activity. 

Despite the ease of the technique and the capability to load nearly every drug by modulating the pore size and duration, electroporation remains on a laboratory scale and appears to be unsuitable for large-scale application. 

## 4. Erythrocyte Membrane as a Carrier for NAs: State of the Art, Advantages, Potential and Drawbacks

Despite being very promising drug carriers, due to their natural role as actual carriers only a few RBC-based formulations are in clinical trials. These include RBC-encapsulated asparaginase (Erytech, Phase 3) and Kan101 (Anokion, Phase 1). Table 2 lists all the ongoing clinical trials based on erythrocyte membrane processing. There is only a small number because of technical difficulties with loading, compatibility in blood type for donor-recipient when autologous loading is not possible, a lack of characterized proteome and interactome for RBCs, the low quality of the preparations, difficult standardization, and a low encapsulation efficiency. After an initial sharp increase in papers on the topic in the 1960s and 70s, research largely stopped after the HIV outbreak [132]. During the period to 2020, on the other hand, the DDS field focused on polymers, dendrimers, antibody/aptamer conjugated artificial nanoparticles, and liposomes, neglecting RBCs. However, the number of publications grew steadily. After the emergence of companies such as Erydel, Erytech, publicly funded research again took an interest in this topic [98]. Interestingly, not even after the RNA interference discovery [133], and the opening up of new frontiers for gene therapy [134], was there an increase in the tentative loading of NAs, neither for transfection purposes nor for gene therapy. This is surprising, because, being largely devoid of RNA molecules, they do not pose a problem with horizontal gene transfer. 

Erythrocyte ghost generation is a well-known and optimized process, but their use as a DDS has a number of drawbacks. Among them, reasons why they have not yet been used for NA delivery can be traced back. 

### 4.1. A Stealth Erythrocyte Ghost: Antigen Compatibility Issue

Autologous blood should be used to avoid allergic reactions. Therefore, the loading procedure must have patient compliance and a scaled-up loading system. Allogenic blood administration is subject to the AB0 compatibility system and the Rhesus antigens (Rh) problem, present as polypeptides inside the RBC membrane (probably ionic channels, codified by adjacent loci [135]). Recently, Zhao et al. solved this problem [136], caused mainly by the most immunogenic antigen, RhD, by engineering a RBC membrane to become, via an immobilized horseradish peroxidase, shielded by a tyramine-polysialic acid 3D hydrogel structure. Therefore, safely administering the subsequently modified erythrocytes in the bloodstream. Previously, the same results for antigen D were achieved with PEGylation [137]. The immunological attenuation for the AB0 antigen system (present in erythrocytes glycocalyx) is traditionally achieved with PEGylation. The RBC ghosts are surface decorated with PEG, usually on lysine residues of Band-3 protein [138,139,140]. The resulting stability and susceptibility to lysis is very dependent on the coupling [97] and linking [141]. In addition, methoxy-PEG [142] is used. Camouflage has been used to decorate the RBC surface using maleimidophenyl-PEG via iminothiolane-mediated thiolation [143,144] or poly-dopamine [145]. Unfortunately, anti-PEG antibodies, IgM and IgG, have been detected in the adult population [40]. This could lead to phenomena like accelerated blood clearance [43], thus totally nullifying the aim of encapsulation, that is, to extend the circulation time. 

### 4.2. Erythrocyte Membrane, Ghost Shape and Permeability: Is It Suitable for NAs Delivery?

#### 4.2.1. Shape

Antigen compatibility leads to another issue involving the maintenance of flexibility, elasticity, and the shape of RBC ghosts. They are naturally deformable entities, but modifications to the surface reports denied any substantial difference between the regular and modified RBCs. Their rheology was intensively investigated for nearly forty years [146,147], and still thriving with the advancements in microfluidics such as “lab-on-chip” approach [148,149]. From a technical point of view, shape and rheology characterization could help scaling up RBCs processing for heterologous applications, speeding up quality assessment of starting material, a step that is currently neglected and that deserves attention.

It is however widely known that shape is a key factor in DDS design [150,151,152,153], in terms of porosity rather than symmetry [154]. As a thumb rule, irregularly shaped or extreme-aspect-ratio particles are less prone to opsonization and subsequent macrophages uptake. With increasing symmetry (i.e., cylindrical and then spherical) cells capable of phagocytosis show higher degrees of internalization and adhesion to the nanoparticles. Furthermore, sharp-edged, high-aspect-ratio nanoparticles easily achieve endosomal escape [155]. 

**Figure 2:**
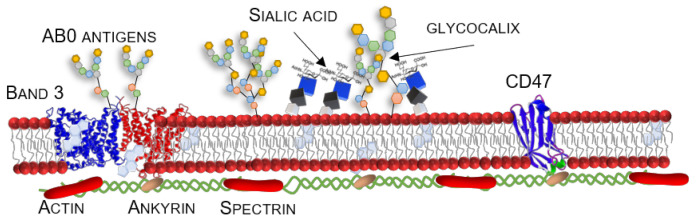
Native erythrocytes and ghosts are features that must be taken in account while developing a drug carrier. Band 3 protein (4YZF [88]), a bicarbonate/chloride ion exchanger, in contact with cytoskeleton via ankyrin, shows on its surface the AB0 antigen glycans; glycocalyx also comprises sialic acid, a phagocytosis mediator or inhibitor; CD47 (2JJS, [84]) is an ubiquitous integral protein that acts as a switch for macrophages phagocytosis interacting with a SIRPα receptor.

Curvature and shape also influence cell fusion [156], a process in which the membranes of two cells come into contact, first via an hemifusion (the close approach of leaflets) and later, membrane budding and conjoining. The erythrocyte membrane, rather than via the traditional PEG-mediated mechanism [157] (where PEG is able, via a still not totally clear mechanism, to help fusion of bilayers [158]), is able to fuse with other erythrocytes via a fusogenic electric pulse, by disturbing the spectrin network (Figure 2) [159]. Their fusogenic pathways are, therefore, SNARE independent [160]. Carrier-target-cell fusion is an important step in therapeutic RNA delivery, especially with exosome mimicking liposomes [161,162]. This can happen via many mechanisms, such as phagocytosis, pinocytosis, endocytosis, lipid-rafts mediate fusion, and tunnelling nanotubes [163]. How the erythrocytes’ surface properties can be optimized is discussed in Section 4.3.

The erythrocyte’s shape is approximatively biconcave. When depleted of Hb (i.e., ghosts) it is still a matter of debate whether they keep, in an isosmotic medium, such a shape, since it is known that, having an ultracomplex membrane composition and a unique cytoskeleton network, their morphology is very dependent upon electrolytes [164]. Relying mainly on the actin-spectrin network, as shown by [165], such a shape is also maintained in ghosts. Furthermore, it is still not known whether the hyperosmotic resealing of the erythrocytes affects the subsequent shape. A stomatocyte is a transient form, cup-like, as opposed to echinocyte, with the latter showing remarkable spiculations on the outer leaflet in SEM investigations [73,166]. Such behavior was partially explained by the so-called “bilayer-couple” effect, in which the more charged inner leaflet causes distortion of the membrane folding [167]. More recent insights showed that this is due to the phosphatidylserine and phosphatidylethanolamine translocation and that the process is reversible when elicited by electroporation in the presence of ATP [130]. It is also caused by a chlorpromazine treatment [73,168]. 

In a few examples of erythrocyte used as a transfection agent [101,169,170] and loaded with NAs, the Sendai virus was first used as a fusing agent by the recipient cells to carry over the “micro-injection”, and later the PEG coating and electrostimulation (see Section 3.2). The mechanisms underlying the membrane fusion were later elucidated and in theory could be applicable to the targeted release of NAs from RBC ghosts, with cell-environment-compatible approaches [160].

Shape is a crucial parameter from the pharmacological point of view. However, erythrocytes have not been characterized in this way. It is also not known whether such processes are ATP-driven, or for how long the discoidal shape is retained in its absence. It is not even known whether aberrant forms such as spherocytes and echinocytes are somehow immunogenic and inflammatory, and whether the deformed membrane can cause early drug diffusion. In order to optimize a drug carrier, and to load it with drugs that are chemically different from what was already used, it is important to explain its overall interaction in vivo and in contact with different target tissues. 

#### 4.2.2. Permeability and Drug-Release Mechanism

There is a lack of specific pharmacokinetic characterization for the permeability and diffusion rates of the RBC membrane with respect to foreign molecules, whether it is acidic, basic, or zwitterionic. However, the ion-induced exchange of drugs between the environment and the erythrocyte is well documented [171,172]. Conversely, the semi permeability (i.e., selective permeability to different solutes) properties of the RBC membrane, dependent upon pore formation and lipidic composition, loaded with NAs of variable molecular weight are totally unknown, in terms of mechanism and kinetics. Nevertheless, it seems that loaded DNA is not released, in vitro, in 24 h [102]. It is known, however, that an increase in cholesterol and in surface protein glycation reduces the diffusion rates [58,173,174,175]. 

It should be pointed out that all the in-trial erythrocyte-based formulations (for example GRASPA^®^, asparaginase loaded in erythrocytes ghosts) contain a systemically working agent (i.e., an enzyme or a glucocorticoid), while an mRNA (for vaccines, cancer immunotherapy or heterologous expression), a siRNA and an anti-sense RNA (for gene therapy) have to be delivered straight to the target, avoiding any contact with the host tissues. Early drug diffusion (i.e., to achieve a controlled release) outside the carrier has to be avoided [176]. This could be by complexing the NA drug with nanoparticles [177,178], lipocomplexes [179] or encapsulating the active ingredient in prodrug form [116]. A glutaraldehyde treatment on a protein cytoskeleton ought to be used only in extreme cases [57]. Traditional, circulation, half-life increasing techniques, such as PEGylation or conjugation with albumin [180], can be applied to the drug [181]. Furthermore, more advanced approaches have been attempted to delay and control the drug release, for instance, through a mellitin-cobalamin hemolytic complex (light triggered) [182]. To use whole erythrocytes as a DDS in the case of gene therapy, RNA vaccines or transfection agents, will need further studies and improvement to build a reliable model comprising blood interactome, RBC proteome, and erythrocyte membrane vs. tissue membrane interactions to ensure maximum delivery timed capacity. Furthermore, natural vesiculation could also be exploited as a spontaneous sustained delivery mechanism, but more studies are needed. 

### 4.3. Improvement of Erythrocyte-Based Drug Carriers: Nano-Erythrosomes

Cell-based therapy is promising, but there are many challenges to be faced when an established system needs adapting to a different drug. All the knowledge described in the previous section has led, as a further step in the biomimetic DDS field, to the generation of nano-erythrosomes [183,184]. Nano-erythrosomes, whose advantages and drawbacks are summarized in Table 3, can be prepared via shear-stress processes, such as sonication and the subsequent extrusion of ghosts up to 200 nm of filter size exclusion [176]. The Deàk group [185,186] characterized the rheological properties of nano-erythrosomes relative to erythrocyte ghosts. No significant protein denaturation occurred during the sonication/extrusion process. The thickness of the overall bilayer is around 11 nm (SAXS data), while for a native erythrocyte it is around 50 nm and 20–25 if compressed up to 10^3^ Pa [187]. The samples are relatively inhomogeneous in terms of hydrodynamic radii. The lipid bilayer is 4 nm thick, while the rest is a glycocalyx forest and cytoskeleton, somewhat deformed by the shear-stress processing. Most of the peripheral proteins are then washed out, while the ones necessary for the stealth effect remain [186]. The Lejeune group reported the absence of in vivo toxicity [184]. Furthermore, the hypotonic swelling of ghosts, sonication and extrusion, do not significantly affect the membrane marker loss such as CD47 and TER119 [188]. So far, nano-erythrosomes have been used as antigen carrier for immune vaccines directed to the spleen [189], daunorubicin-decorated [184], for fasudil encapsulation [190], indocyanine green loaded [191,192], as a stealth coating for an *Escherichia coli*-based microswimmer [188], and loaded with iron-oxide nanoparticles for magnetic resonance imaging [193]. The CD47 features are advantageous if a macrophage intracellular delivery is desired (for instance, in the case of HIV infected macrophages). For healthy erythrocytes, it usually works like “don’t eat me” markers for macrophages, that is, in senescent or unhealthy cells it undergoes conformational changes, binding other co-factors, thus acting as a promoter of phagocytosis by RES effectors [78]. 

A nano-erythrosome suspension can also be described with colloidal matter properties, like for other drug-delivery nanoparticles, such as zeta-potential, which is similar to native erythrocytes, that is, around −20/−42 mV [185,194], due to syalil moieties that are present. Similar to liposomes, the disturbance of the order of acyl tails in lipids can be measured. This was shown to be reversible up to 50° [195]. Therefore, a suspension could be characterized using techniques such as DLS, SAXS, SANS, freeze-fracture/liquid cell TEM, 1D-NMR, Fourier-transformed IR and Raman spectroscopies. It should be pointed out that the biconcave shape is still retained in nano-erythrosomes when extrusion is avoided [186,194]. Such an irregular shape could be advantageous as a DDS for endosomal escape [155]. As for parental erythrocytes, such a phenomenon is probably due to the Canham-Helfrich effect [196,197]. Moreover, at the phase-transition temperature for membrane lipids (50 °C), they exhibit a reversible but remarkable polygonal arrangement of spectrin-actin-ankyrin scaffold upon dipalmitoylphosphatidylcholine (DPPC) doping [186], usually found in clathrin-coated vesicles. Furthermore, as colloidal matter, they are not prone to coalescence due to electrostatic repulsion and behave in a suspension as nearly undistinguishable from uni-lamellar vesicles such as coated of PEG_2000_ [193,194]. The composition of nano-erythrosomes, in terms of lipids and integral membrane proteins, is superimposable on that of erythrocyte extracellular vesicles (exosomes and micro-vesicles altogether) [198,199], while the latter lack external cytoskeleton, being originated by events of vesiculation [200]. We can speculate that most of the effects studied for EV and applications are also adaptable, at least avoiding mechano-elastic implication, to nano-erythrosomes.

#### 4.3.1. Nano-Erythrosomes: Clearance and Endocytic/Phagocytic Pathways

**Figure 3:**
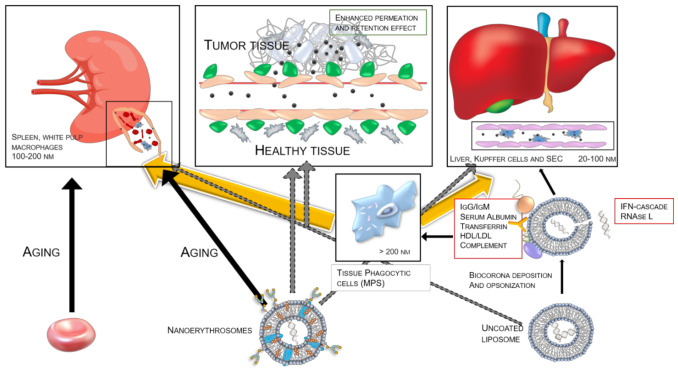
Main clearance pathways of erythrocytes, nano-erythrosomes and uncoated liposomes (i.e., without PEGylation). Solid black arrows represent the majority fate of the nanoparticles. In yellow arrows, the rapid and unescapable clearance exerted by spleen and liver on nanoparticle after mononuclear phagocyte system recognition. Dashed arrows show the minority clearance pathways. In the interaction with mononuclear phagocyte system, there is a strong dependence on the opsonization of nanoparticles, by antibodies, serum albumin, etc. If the nanoparticle is loaded with dsRNA, the sensing of such a presence elicits the activation of interferone-tyrosin kinase cascade [4] and RNAse L activity. Then, the liposome is degraded by the liver. In the case of nano-erythrosomes, the supposed clearance pathways partially superimpose on the liposomes. However, being recognized as self, they are not be prone to MPS interaction. The most wanted localization of a nanoparticle in cancer therapy is in tumor tissues. Nanoparticles have to be small enough to take advantage of the EPR effect [27] Lastly, the natural process of aging in erythrocytes leads to their degradation in the spleen. The spleen model can be retrieved here (bit.ly/3uMnhsl)*e* for attribution.

Nano-erythrosomes, while retaining erythrocyte stealth, biocompatible, atoxic, and efficient carrier properties, have the advantages of a liposomes-sized particle. Traditionally, according to the ADME rules, the tentative resolution of the “distribution” rate problem has focused on RES macrophages residing in the liver, bone marrow and lymph nodes, and hepatic clearance (by Kupffer cells). Conversely, a recent pharmacological problem is that of spleen clearance [202,203]. In normal conditions and no surface-functionalization, erythrocyte-sized nanoparticles are cleared by the spleen in the red pulp. Being a highly perfused organ (170 mL/min/100 g), and composed by the endothelial cells, having little or no capacity of storage, the impact of a nanoparticle on the spleen is basically on the white pulp, colonized mainly by B lymphocytes and marginal-zone macrophages. It was determined [202] that size has a dramatic influence on the fate of a nanoparticle in the human body. However, for entities like liposomes or colloidal nanoparticles of 100–200 nm, the main fate after intravenous delivery is the uptake from splenic macrophages in the white pulp. For larger hydrodynamic radii (up to 400 nm) the filtration is mainly on the red pulp and it accounts, in a few hours, to nearly the whole of the administered dose [153]. The lesser known system of Scavenger Endothelial Cells (SEC) [204], mainly residing in the liver sinusoids, competes with RES for the nanoparticles’ uptake. Nanoparticle clearance from the bloodstream is clathrin-mediated, not phagocytic, for 70-nm silica nanoparticles, while no data are available about liposome-like objects [205]. Therefore, size-reduction processes such as extrusion and sonication also delay the clearance of the DDS from the blood. An optimal size, to avoid opsonization and immune-system recognition in blood and tissues [206], albumin, or HDL/LDL deposition in blood [153,207], IgM binding [208] and to minimize splenic, hepatic, and renal clearance [43,206], would be 100–200 nm. It should be recalled that the term reticuloendothelial system (RES) refers to a various and scattered population of phagocytic cells of the liver, bone marrow, lymph nodes, spleen, brain microglia, and each population and phenotype has a different optimum nanoparticle uptake [38]. MPS (mononuclear phagocytic system) is a broader definition for phagocytic/endocytic cells, also residing in blood and tissues [209], which help clearance organ RES to get rid of the nanoparticles (Figure 3).

Uptake by phagocytic cells, composing MPS and RES, could be also avoided or at least reduced, for nano-erythrosomes, by the lack of formation of the so-called “biocorona”. This was discovered and studied by the Vroman group in the 1960s [210,211] (Figure 3). The complement protein, apo-lipoproteins, opsonins, albumin, thus not masking the presence of CD47 and TER119 and, most importantly, subsequently eliminating the need for PEGylation, chitosan, pluronic [49] or dextran coating, that is, branched polymers known to elicit several immune responses [43,212]. Moreover, while the fenestrations of normal arteries range from 5 to 100 nm in diameter [203], in the case of defective blood vessels in cancer tissue [213], known to be leaky, the pores can reach 2 µm. This leads to the EPR effect [27], exploited in cases of liposome-based formulations in passive tumor targeting. The natural origin of nano-erythrosomes, and, therefore, the lack of biocorona formation, will help reduce the total clearance. It was determined by the Maeda group in 1986 that protein and polymer complexes are more efficiently accumulated in tumors and that liver and spleen clearances are dramatically lower [214]. In any case, RES phagocytosis could also be desirable sometimes, such as in HIV-infected macrophages [106,215]. Internalization and degradation processes can be accelerated by irreversible clustering of band-3 protein via a zinc chloride treatment [216], causing IgG opsonization, that can be extended to nano-erythrosomes. It has yet to be determined whether nano-erythrosomes retain CR1 (complement receptor 1) as parental erythrocytes, a complement complex receptor known to cause, upon recognition of opsonized particles, a conformational change in surrounding lipid double layer. If this is the case, how this will affect nano-erythrosomes behavior in vivo [78].

In the case of NA loading, that is, as gene-therapy agents, the delivery of the cargo must be cytoplasmatic. Miniaturization could also improve the endocytosis and internalization. Liposomes are usually internalized by a cell via endosomes, phagocytized, or fused with an outer bilayer, releasing the cargo within the cytoplasm [217]. The pH resistance of nano-erythrosomes is also unknown. Thus, so is their ability to release the payload when in contact with endosomal acidic pH. Due to the similar size, it can be assumed that nano-erythrosomes too, despite the stiffness of the membrane due to the presence of cholesterol, can take advantage of similar pathways. So far, even if in presence of precise hints on the internalization of nanoerythrosomes by cancer cells and endothelial cells [192,218], the mechanism is still unclear.

Therefore, while nano-erythrosomes show features belonging to both erythrocytes and liposomes, they have not been used for transfection or gene therapy.

#### 4.3.2. Tissue Penetration of Nano-Erythrosomes in Solid Tumors and Active Targeting

A commonly neglected problem for the in-vivo application of liposomes is their tissue penetration, that is, the ability to reach deep tumor regions, far from main blood vessels. A nanoparticle, to exert its payload release activity, has to cross several barriers: (1) to reach the tumoral blood vessels; (2) to extravasate from them, and pass through epithelia; (3) to survive the journey inside the extracellular matrix and the stroma and come into contact with tumor cells; (4) to make it to every region of tumor tissue, via intercellular or interstitial transport and, finally, to release its payload [219]. Solid tumor cells are usually far from blood vessels, the latter being compressed and less efficient for nutrient and oxygen transportation [220]. Therefore, anaerobiosis and autophagy are efficient tumoral markers [221]. A huge challenge in nanomedicine for gene-therapy applications is to reach the stem tumoral cells, even more fundamental than being internalized by the cells. The activity of therapeutic NAs is independent of the cell cycle stage (often crucial for traditional chemotherapy), the limiting step is the diffusion and drug release by carriers [222], hampered by the dense extracellular matrix that the tumor cells are surrounded by. The microfluidic of the neoplastic blood vessels is also distorted by the high pressure exerted by abnormal cells and the lack of lymph drainage. No coherent model for liposomal nanoparticle behavior in such vessels has been developed [223], although there have been some attempts to find a route to better tumor penetration [224]. A bio-mimetic DDS, such as nano-erythrosomes, can better dissolve within the collagen extracellular matrix, due to being partly protein composed [214]. The presence of metalloproteinases in the ECM of some solid tumors [225] can also improve the drug release from nano-erythrosomes, degrading the membrane proteins and disrupting the membrane structure. 

Active targeting can also improve tissue penetration, as it is a common technique in DDS design [226,227]. The erythrocyte surface has been decorated with multiple agents, for example, via biotin-avidin coupling, lipid fusion, amino acid binding [97,228,229]. A similar approach was used to conjugate RBC-derived membranes, when used as a cloaking agent for nanoparticles, with antibodies [230]. Antibody conjugation has also been exploited for nano-erythrosomes. While loaded with indocyanine green for near-infrared fluorescence imaging, they are conjugated with anti-HER2 (human epidermal growth receptor) antibodies via DSPE-PEG-NH_2_ (2000 Da in molecular weight), to achieve better in vivo imaging properties [192]; also, they have been functionalized with ephrin-B1 ligand receptor, to actively target human dermal microvascular endothelial cells [218]. 

### 4.4. Possible Bottleneck: The Encapsulation Efficiency of NAs

There are only a few examples of NA-encapsulated erythrocytes (or derived objects). Such a process poses challenges. The main aim of encapsulation is to protect the NAs from the body, which, in the case of contact with the naked, double-stranded RNA, reacts violently by activating interferone-tyrosin-kinases cascade [4]. The second aim is to deliver the cargo directly into the recipient cell, via a fusogenic or endocytic pathway. That has to be elicited somehow, and the effectiveness of this step determinates the transfection efficiency. Antisense oligonucleotides, where approved, such as Eteplirsen against Duchenne muscular atrophy (an only FDA, not EMA, approved orphan drug) have to be modified to avoid cleavage by nucleases, in the form of phosphorodiamidate morpholino oligomer [231], or, as Prexigebersen (phase II, NCT02781883), have to be microencapsulated. In RNA-interference-based therapy, the reaction against dsRNA can be avoided by administering a DNA strand codifying for the relative short-hairpin RNA [232]. In a drug’s scaling-up process, the first challenge is the efficiency of encapsulation of the drug inside the carrier. Such a process is regulated by the loading method, charge, carrier size, state of aggregation of the drug, and the lipid composition if dealing with liposomes [233,234]. While for weakly alkaline drugs the remote loading (and entrapping) technique based on the transmembrane ammonium sulphate gradient can be used, the same cannot be applied to NAs [235]. In other words, there are no chemical routes to follow to enrich and entrap a NA, unmodified, in a carrier. The negative charge of NAs has been used with the development of cationic liposomes for gene therapy. For example, SGT-53 (NCT02340156) is a regular p53 (a famous oncosuppressor gene) cDNA, entrapped in a DOTAP/DOPE liposome decorated with a targeting antibody. In such structures, the NA is condensed with a lipidic core, in lipoplexes [179]. Otherwise, a solid lipid nanoparticle (not hollow) has been developed to bind NAs, thus controlling their release kinetics: EnCore, currently used to entrap MYC oncogene ASO [236]. 

Nano-erythrosomes and parental erythrocytes show a negative surface charge, due to the sialic acid moieties. Theoretically, this will impair the encapsulation efficiency of the NAs. Therefore, a possible solution could be the use of DNA block copolymers, neutralizing the NA’s negative charge by complexing the DNA with an hydrophobic polymeric core [237]. Conversely, the use of “helper lipids”, developed for cationic nanoparticles, could improve the transfection efficiency for nano-erythrosomes, customizing the chemical composition according to the target tissues [238]. The lipid-fusion-loading technique can be used to load erythrocytes and nano-erythrosomes with NAs. 

The negative charge of NAs leads them towards cationic residues such as lysine and arginine, as shown by their adsorption in L-lysin dendrimers [239,240]. The extent of such binding, if somehow sterically impaired by the presence of the glycocalyx, is unknown. mRNA is, however, known, in some exceptional cases, to insert permanently into DOPC double layers [241,242], Such studies have never been carried out for homologous in sequence DNA. Therefore, a mix of electrostatic repulsion between the surface sialic acid moieties and the NA, the adsorption in the glycocalyx forest, the interaction with surface proteins, and, lastly, the eventual insertion of the RNA in the double layer, will hamper the loading efficiency of erythrocytes and nano-erythrosomes. Therefore, an accurate selection based on molecular charge and overall hydrophobicity surface must be carried out for a NA if it is to be loaded into RBCs, while a nuclease enzymatic treatment could show the binding localization on the outer surface. 

## 5. Conclusions and Future Perspective

Erythrocytes have long been used as DDSs, but only recently began to attract attention outside the academic community. This is thanks to the approval of some erythrocyte-based technologies such as the proposals of Erydel and Erytech, while GRASPA (L-asparaginase encapsulated in RBCs) did not make it to the end of the trial-II process. Erythrocytes could provide, without any further modification, everything a DDS needs: stealth ability to deceive the immune system, protection against proteases and nucleases present in the blood, a long half-life in the latter, leading to longer therapeutic effects. They are easy to collect and administering the patient with self-erythrocytes loaded with the drug could avoid any allergic reaction. Among the drawbacks, there is the not-fully-characterized rheology of the membrane, in terms of shape, porosity, permeability, and supramolecular behavior, from a pharmacokinetic point of view, especially when trying to use erythrocytes as carriers for NA-based drugs. The latter are currently a topic in pharmacology, due to the discovery of RNA interference and the recent advancements in gene therapy. Only a few examples could be counted for the applications of erythrocytes for the previously mentioned molecules, while the whole topic, also thanks to the development of Sars-Cov-2 mRNA vaccines, continues to thrive. The recent “nano revolution” in pharmacology has reached the declining field of erythrocyte research too: extracellular vesicles, with sizes ranging around 100–200 nm, also from erythrocytes, have begun to be a subject of interest. Furthermore, in the late 1990s, nano-erythrosomes were developed from the sonication and extrusion of RBC ghosts. They retain the stealth abilities of erythrocytes, but their reduced size allows them to circumvent clearance issues, while permitting a widespread distribution of the loaded drug. All the knowledge acquired along decades about erythrocyte membrane and cytoskeletal proteins could also be applied to them. While they have been fully characterized from a colloidal matter point of view, biological data about the interaction with tissues, cell fusion or phagocytosis, eventual endosomal escape, and, finally, stability when drug-loaded, are still lacking. They could be an interesting platform to develop new-generation NA-based drugs that are stealthy and autologous. 

## Figures and Tables

**Table 1:** Comparison of techniques and cases of erythrocytes loaded with NAs.

Loading Technique	NA Loaded	Encapsulation Efficiency	Pre-Treatment of n.a.	Integrity of NA	Reference
Hypotonic swelling	Plasmidic and genomic DNA	20%	Compacted with PEG_6000_	Not reported	[100]
Hypotonic swelling and freeze-thaw cycles	DNA for transfection	6–20%	None	Not reported	[101]
Hypotonic swelling	Anti-sense Oligonucleotides	10%	Complexed with 25 kDa polyethyleneimine	Not reported	[102]
Hypotonic swelling	tRNA	10%	None	Around 50% on PAA gel	[103]
Electroporation	mRNA on whole blood cells	No quantified	None	No data	[13]
Hypotonic swelling + electroporation	Plasmidic DNA	Not quantified	None	Integer and amplifiable with RT-PCR	[5]
Isotonic dialysis	RNA and DNA	Up to 35% with 37° incubation; larger for smaller molecules	None	Integer as assayed on gel-electrophoresis	[104]
Hypotonic dialysis	Antisense Peptide NA	14%	None	Assumed integer as assayed by HPLC	[105]
Hypotonic dialysis	Antisense Peptide NAs	Around 10%	None	Assumed integer as assayed by HPLC	[106]

**Table 2:** Erythrocyte-based formulations in clinical trials. All URLs were accessed in 10 May 2021.

Condition Treated	Drug	Company	Trial Number
Ataxia telangiectasia	Dexamethasone 21-phosphate	EryDel Italy & USA http://www.erydel.com	NCT02770807
Acute lymphoblastic leukemia/pancreatic cancer	Asparaginase	ERYtech Pharma France and USA http://www.erytech.com	NCT02195180
Mitochondrial neurogastrointestinal encephalomyopathy	Thymidine phosphorylase	St George’s, University of London UK The Clinical Trial Company UK Orphan Technologies Ltd. CH	NCT03866954
Phenylketonuria	RTX-134	Rubius USA http://www.rubiustx.com	NCT04110496
Celiac disease	KAN101	Anokion USA & Switzerland https://anokion.com	NCT04248855

**Table 3:** Summary of established advantages and possible drawbacks of nano-erythrosomes use as drug carrier.

Properties	Advantages	Possible Drawbacks
**Interaction with blood plasma**	Virtually no opsonization, complement activation, protein deposition, IgG or IgM interaction in mature and normal erythrocytes	Low immunogenic effect on innate immune system cells without any further functionalization if used as DDS for epitope vaccination; Unknown, if any, composition of the “biocorona” of nanoparticles
**Accumulation and clearance**	Assumed being able to cross leaky blood vessels fenestration and tendency to accumulate in cancer tissues due to EPR effect.	Extravasation could lead to a-specific delivery Phosphatidylserine exposure on outer leaflet could lead to rapid clearance; Due to the size of hydrodynamic radius (100–200 nm), there could be still uptake from spleen MZ-macrophages and hepatic Kupffer cells; Unknown mechanisms of internalization in cells, of endosomal capture and escape (if any)
**Drug delivery and release mechanism**	Possible internalization similarly to liposomes: bilayer fusion, endocytosis and endosomal escape, phagocytosis.	Unknown solubility or partition of a random drug within the bilayer or in the hollow core; Unknown kinetic of model-drug release;
**Surface modifications**	Huge yield in terms of surface/volume ratio, 1000–2000 nano-erythrosomes of 200 nm from one single erythrocyte; Easy surface decoration for multiple purposes due to the presence of membrane proteins retained (targeting, PEGylation, polymer coating)	External loading with organic nanoparticles could lead to merging ROS generation and subsequent lysis [201]
**Stability**	No known tendency to coalesce and aggregate, due to electrical repulsion among nanoparticles; Thermodynamic stability even after freeze drying [185,186] No need for surface stabilizing agents.	In absence of ATP unknown kinetic of flip flop of lipids; Unknown pH resistance; Possible mechanic and osmotic fragility due to hemoglobin depletion and shear stress treatments.
**Drug loading efficiency**	In isotonic condition, polarity and solubility in water of the drug are not conditions affecting the loading efficiency; Embedding the drug in the bilayer and in the glycocalyx is possible.	Eventual binding of the drug to inner-membrane of the nano-erythrosome must be characterized; Uncertainty of which loading method is the most suitable; Remote active loading techniques cannot be used [28]; Electric repulsion between carrier and drug in case of NAs; Possible unwanted external loading of NAs on surface proteins

## Data Availability

Not applicable.

## Abbreviations

DDS	drug delivery system
RBC	red blood cell
RBCM	red-blood-cell membrane
Hb	hemoglobin
NP	nanoparticle
NA	nucleic acid
EV	extracellular vesicle
SNARE	soluble N-ethylmaleimide-sensitive factor attachment receptor
PAA	poly-acryl-amide
ROS	reactive oxygen species
DOTAP	N-[1-(2,3-Dioleoyloxy)propyl]-N,N,N-trimethylammonium methyl-sulphate
DOPE	dioleoylphosphatidylethanolamine
RES	Reticulo-endothelial system
MPS	Mononuclear phagocyte system
SEC	Scavenger endothelial cell
ASO	antisense oligo-nucleotides
PGLA	poly(lactic-co-glycolic acid)
ECM	extracellular matrix
